# CPK2 Enhances ABA Sensitivity in Seed Germination and Root Growth by Promoting ABA-Induced *ABI5* Expression and ABI5 Protein Stability

**DOI:** 10.3390/plants14172671

**Published:** 2025-08-27

**Authors:** Xiaoju Liang, Wei Zhu, Weifeng Xu, Jiansheng Liang

**Affiliations:** 1 College of JunCao Science and Ecology, Fujian Agriculture and Forestry University, Fuzhou 350002, China; liangxiaoju718@163.com; 2 Shenzhen Key Laboratory of Plant Genetic Engineering and Molecular Design, Institute of Plant and Food Science, Southern University of Science and Technology (SUSTech), Shenzhen 518055, China; zhuw@scau.edu.cn; 3 Department of Biology, School of Life Sciences, Southern University of Science and Technology (SUSTech), Shenzhen 518055, China

**Keywords:** ABA, ABI5, CPK2, root growth, seed germination, *Arabidopsis*

## Abstract

Abscisic acid (ABA) is a crucial phytohormone that functions as a master regulator of plant growth and development, as well as responses to diverse abiotic stresses, by integrating environmental cues with developmental programs. The transcription factor ABA INSENSITIVE 5 (ABI5) functions at the central hub of the ABA signaling pathway and mediates the expression of its target genes. Emerging evidence reveals extensive crosstalk between calcium-dependent protein kinases (CPKs)-mediated calcium signaling and the ABA-ABI5 cascade, enabling plants to balance growth and stress responses. However, the molecular mechanisms underlying the interactions between CPKs and ABA-ABI5 signaling are still elusive. In this study, we revealed that CPK2 enhances sensitivity to ABA during both seed germination and seedling root growth by promoting ABA-induced *ABI5* expression and increasing ABA-mediated ABI5 stability. Compared to the wildtype (Col-0), the *CPK2*-OE line exhibited the highest sensitivity to ABA in both seed germination and root growth, while the *cpk2abi5-7* double mutant showed the least sensitivity. The single mutants *cpk2* and *abi5-7*, as well as the *abi5-7CPK2-OE2* line, displayed intermediate phenotypes, suggesting that CPK2 acts upstream of ABI5. Biochemical and molecular biological studies revealed that CPK2 physically interacts with ABI5 and directly phosphorylates it at Ser42, Ser145, and Thr201. Moreover, both ABA-induced *ABI5* expression and protein accumulation were significantly reduced in *cpk2* mutants upon ABA treatment. Taken together, these findings provide compelling evidence that CPK2 exacerbates the ABA inhibition of seed germination and root growth by enhancing both the expression and stability of ABI5, thereby reinforcing stress adaptation during early plant development.

## 1. Introduction

Abscisic acid (ABA) is a central phytohormone that orchestrates a wide range of physiological and developmental processes by integrating environmental stimuli with intrinsic developmental programs, including seed dormancy, germination, early seedling development, and stomatal closure [1,2,3,4]. The influence of ABA on these processes, as well as plant responses to environmental cues, is deeply intertwined with its signaling pathways and biosynthesis dynamics [5,6,7,8].

Over the past two decades, the core ABA signaling pathway has been well documented. It involves PYR/PYL/RCAR receptors that inhibit PP2C phosphatases, leading to SnRK2 kinase activation. Once activated, SnRK2s phosphorylate and activate downstream transcription factors, particularly those belonging to the basic leucine zipper (bZIP) family, such as ABA-responsive element-binding factors (ABFs) and ABA INSENSITIVE5 (ABI5), which are essential for triggering ABA-responsive gene expression [9,10,11,12].

ABA INSENSITIVE 5 (ABI5), a bZIP transcription factor preferentially expressed in dry seeds and strongly induced by exogenous ABA, plays a critical role in ABA-mediated inhibition of seed germination and post-germinative growth in *Arabidopsis* [13,14,15,16,17,18,19,20,21]. Genetic studies have shown that *abi5* loss-of-function mutants are hyposensitive to ABA, whereas plants overexpressing ABI5 display hypersensitivity [13,14,15,22]. As a transcription factor, ABI5 regulates the expression of numerous target genes involved in stress adaptation [20]. Previous studies have identified thousands of ABI5-targeted genes [23,24,25,26,27]; however, the detailed functions of these genes in ABA-regulated seed germination and root growth remain elusive.

Calcium-dependent protein kinases (CPKs) represent another critical class of regulators in ABA signaling in *Arabidopsis*. As calcium sensors, CPKs integrate stress-evoked Ca^2+^ signals into cellular responses, linking upstream Ca^2+^ fluctuations to downstream physiological changes [28,29]. Structurally, CPKs are Ser/Thr protein kinases characterized by an N-terminal kinase domain fused to a C-terminal calmodulin-like domain, which maintains the enzyme in an auto-inhibited state until activated by Ca^2+^ binding. The *Arabidopsis* genome encodes 34 CPKs, many of which have been implicated in ABA-mediated processes such as stomatal movement, seed germination, and drought tolerance [30]. Notably, functional redundancy is a common feature among CPK family members due to overlapping expression patterns and substrate specificities, complicating the genetic dissection of individual roles [31]. For example, CPK3, CPK4, CPK6, and CPK11 positively regulate ABA signaling by phosphorylating key bZIP transcription factors such as ABF1 and ABF4, thereby promoting the expression of stress-responsive genes [32,33,34,35]. In contrast, CPK9 and CPK12 act as negative regulators, modulating ABA sensitivity during stomatal closure and seed germination, respectively [36,37]. Other studies have shown that CPK10 enhances drought tolerance and promotes ABA/Ca^2+^-induced stomatal closure, while CPK21 and CPK23 influence stomatal aperture and hyperosmotic stress responses [38,39,40]. Collectively, these findings position CPKs as central integrators of calcium and ABA signaling, making them promising targets for enhancing plant resilience through biotechnological approaches [31].

CPKs and ABA-ABI5 signaling represent two pivotal regulatory systems that enable plants to balance growth and stress responses. Emerging evidence reveals extensive crosstalk between CPK-mediated calcium signaling and ABA-ABI5 cascades [31]. Despite these advances, the full substrate networks and functional interactions of many CPKs remain incompletely understood. Moreover, the presence of functional redundancy among CPK family members complicates genetic analyses and hinders the precise characterization of individual roles. In this study, we revealed that CPK2, as a novel regulator of ABA signaling, physically interacts with and phosphorylates ABI5, thereby enhancing its stability and transcriptional activity. This CPK2-ABI5 module amplifies ABA signaling and exacerbates ABA-mediated inhibition of seed germination and root growth. Our results provide new insights into the molecular mechanisms underlying ABA-regulated seed germination and root growth and highlight CPK2 as a non-redundant component in the complex CPK-ABA regulatory network.

## 2. Materials and Methods

### 2.1. Plant Material and Growth Conditions

The *Arabidopsis thaliana* Columbia (Col-0) ecotype was used as the wild-type background in this study. The T-DNA insertion mutant *cpk2* (SALK_036166) was obtained from the *Arabidopsis* Biological Resource Center (ABRC). The double mutant *cpk2abi5-7* was generated by crossing the single mutants *cpk2* and *abi5-7* [41]. Homozygous T-DNA insertion lines were confirmed by PCR using primers listed in Supplemental Table S1. To generate *abi5-7CPK2-OE2*, the *abi5-7* mutant was crossed with the overexpression line *CPK2-OE2*. Construction of *CPK2-OE2* transgenic plants is described in detail in Section 2.2.

Seeds were surface-sterilized and sown on Petri dishes containing half-strength Murashige and Skoog (1/2 MS) agar medium (0.8%, *w*/*v*). After stratification at 4 °C for 2 days, plates were transferred to a growth chamber maintained at 22 °C under long-day conditions (16 h light/8 h dark). For soil growth, 7-day-old seedlings were transplanted into nutrient-rich soil and grown in a greenhouse under the same photoperiod and temperature conditions.

*Nicotiana benthamiana* seeds were also grown in nutrient-rich soil under long-day conditions (16 h light/8 h dark) at 22 °C. Three-week-old plants were used for transient transformation experiments.

### 2.2. Generation of Transgenic Plants

To construct the CPK2 overexpression vector, the full-length coding sequence (CDS) of CPK2 (1938 bp) was amplified by PCR and cloned into the binary vector pGWB505 under the control of the CaMV 35S promoter (The primer sequences used are listed in Supplementary Table S1). The resulting construct was introduced into Agrobacterium tumefaciens strain GV3101 and transformed into wild-type Arabidopsis plants via the floral dip method [42]. Homozygous T3 transgenic lines were selected and used for further analysis.

### 2.3. ABA Treatments and Root Length Analysis

For gene expression and immunoblot analyses, 7-day-old *Arabidopsis* seedlings were transferred to 1/2 MS agar plates supplemented with either 10 μM ABA (Sigma-Aldrich, St. Louis, MO, USA) or an equal volume of ethanol (mock control), and samples were collected after the indicated time points for RNA or protein extraction.

To assess root growth sensitivity to ABA, seeds of various genotypes were sown on vertical 1/2 MS agar plates containing 10 μM ABA. Seedlings were grown for 11 days before root length measurements were taken. Images were captured at defined time points for phenotypic evaluation.

For seed germination assays, seeds were sown on 1/2 MS agar medium with or without 0.5 μM ABA. After sowing, all plates were subjected to cold stratification at 4 °C in the dark for 2–3 days to synchronize germination. The plates were then transferred to a growth chamber maintained at 22 °C under long-day conditions (16-h light/8-h dark photoperiod). Germination rates were recorded at 3 days after transfer to the growth chamber.

### 2.4. Split-LUC Complementation Assay

For the luciferase complementation imaging assays, the full-length CDS of CPK2 was amplified by PCR and cloned into pCambia1300-nLUC to obtain CPK2-nLUC. The ABI5-cLUC was previously described [43]. Site-directed mutagenesis was performed on the ABI5-cLUC vector to generate the ABI5^Ser42A^-cLUC, ABI5^Ser145A^-cLUC, and ABI5^Thr201A^-cLUC mutants (The primer sequences used are listed in Supplementary Table S1). Recombinant Agrobacterium strains carrying the corresponding constructs were infiltrated into *N. benthamiana* leaves. After 2 days, 1 mM luciferin was sprayed onto the leaf surfaces, and plants were placed in darkness for 5–10 min. Luminescence signals were then detected using a CCD imaging system (Tanon 6100C, Shanghai, China).

### 2.5. Co-Immunoprecipitation Assay (Co-IP)

To investigate the in vivo interaction between CPK2 and ABI5, the coding sequence (CDS) of ABI5 was cloned into the pGWB405 vector to generate the construct 35S:ABI5-GFP, while the CDS of CPK2 was cloned into the pGWB414 vector to produce 35S:CPK2-HA. These constructs were then introduced into *Agrobacterium tumefaciens* strain GV3101 for transient expression in *N. benthamiana*.

For the Co-IP assay, leaves from three-week-old *N. benthamiana* plants were infiltrated with *Agrobacterium* cultures carrying the appropriate vector combinations. Two days post-infiltration, leaf tissues were harvested, flash-frozen in liquid nitrogen, and ground into fine powder. Protein extraction was carried out using IP buffer (50 mM Tris-HCl, pH 7.5, 150 mM NaCl, 0.5 mM EDTA, 0.5% Triton X-100, supplemented with protease inhibitor cocktail). Samples were incubated on ice for 1 h, followed by centrifugation at 12,000× *g* for 10 min at 4 °C. The supernatant was collected and incubated with anti-GFP monoclonal antibody-conjugated magnetic beads (MBL, Beijing, China) overnight at 4 °C with gentle rotation. The immunoprecipitated complexes were washed three times with washing buffer (50 mM Tris-HCl, pH 7.5, 150 mM NaCl, 0.5 mM EDTA, 0.5% Triton X-100). Both the immunoprecipitated proteins and input extracts were subjected to immunoblot analysis. GFP-tagged ABI5 and HA-tagged CPK2 were detected using anti-GFP (MBL, Beijing, China) and anti-HA (Roche, Basel, Switzerland) antibodies, respectively, each used at a dilution of 1:5000.

### 2.6. Protein Expression and Purification

Recombinant protein plasmids were transformed into *Escherichia coli* BL21 (DE3) cells. A single colony from each transformation was selected and first cultured in 5 mL of LB liquid medium containing the appropriate antibiotics at 37 °C for 12 h. This pre-culture was then transferred to 100 mL of fresh LB medium with corresponding antibiotics and incubated at 37 °C for 2–4 h until the OD_600_ reached approximately 0.6.

Unless otherwise specified, recombinant protein expression was induced by adding 0.1 mM isopropyl-β-D-thiogalactopyranoside (IPTG), followed by incubation at 16 °C for 12–16 h. GST-tagged proteins were purified using GST Mag-Beads (BBI, Shanghai, China, catalog no. C650031), and His-tagged proteins were purified using His Mag-Beads (BBI, Shanghai, China, catalog no. C650033), according to the manufacturer’s instructions.

### 2.7. Pull-Down Assay

For the GST pull-down assay, CPK2 fragments were cloned into the pMAL vector to generate MBP-His-CPK2 fusion proteins. ABI5-GST was previously described [43]. In brief, 2 μg of MBP-CPK2-His protein was incubated with 1.5 μg of ABI5-GST protein and subsequently immunoprecipitated using GST beads at 4 °C for 1 h. The beads were collected using a magnetic stand, washed four times with PBS buffer, and eluted with 10 mM reduced glutathione. The eluted proteins were separated by SDS-PAGE and analyzed by immunoblotting using anti-His (MBL, Beijing, China) and anti-GST (Beyotime Biotechnology, Shanghai, China) antibodies.

### 2.8. In Vitro Phosphorylation Assay

For the in vitro phosphorylation assay, the coding sequences (CDSs) of ABI5^S42A^, ABI5^S145A^, and ABI5^T201A^ were amplified and cloned into the pGEX6p-2 vector to generate ABI5^S42A^-GST, ABI5^S145A^-GST, and ABI5^T201A^-GST, respectively. The CDS of CPK2 was cloned into the pMAL-MBP-His vector to produce MBP-CPK2-His.

In brief, 1.5 μg of MBP-CPK2-His protein was incubated separately with 1.5 μg of ABI5-GST, ABI5^S42A^-GST, ABI5^S145A^-GST, or ABI5^T201A^-GST in phosphorylation buffer (20 mM Tris-HCl, pH 7.5; 5 mM MgCl_2_; 1 mM DTT; 1 mM CaCl_2_; 50 μM ATP; protease inhibitor cocktail; protein phosphatase inhibitors) at 30 °C for 30 min. The reaction was terminated by the addition of SDS loading buffer, followed by heating at 100 °C for 5 min. Reaction products were separated by SDS-PAGE and analyzed via immunoblotting using an anti-phosphoserine/threonine antibody (PhosphoSolutions, Aurora, CO, USA) at a dilution of 1:1000.

### 2.9. RNA Isolation and RT-qPCR Analysis

Total RNA was extracted from plant tissues using the ReliaPrep™ RNA Miniprep System (Promega, Madison, WI, USA) according to the manufacturer’s instructions. For RT-qPCR analysis, first-strand cDNA was synthesized from 1 μg of total RNA using the NovoScript^®^ Plus All-in-one 1st Strand cDNA Synthesis SuperMix (Novoprotein, Suzhou, China). Quantitative real-time PCR (qPCR) was carried out on an Applied Biosystems 7500 Real-Time PCR System using SYBR Green Master Mix (YEASEN, Shanghai, China), following the manufacturer’s recommended protocol. The *Arabidopsis* Actin2 gene was used as an internal reference for normalization. Gene-specific primer sequences used in this study are listed in Supplemental Table S1 or were derived from previous studies [43].

### 2.10. Quantification and Statistical Analysis

Data for quantification analyses are presented as mean ± standard error (SE) or standard deviation (SD). Statistical significance among treatments was examined by Student’s *t*-test, two-way analysis of variance (ANOVA) test (Tukey’s multiple comparisons test).

## 3. Results

### 3.1. CPK2 Enhances the Sensitivity of Root Growth to ABA

**Figure 1:**
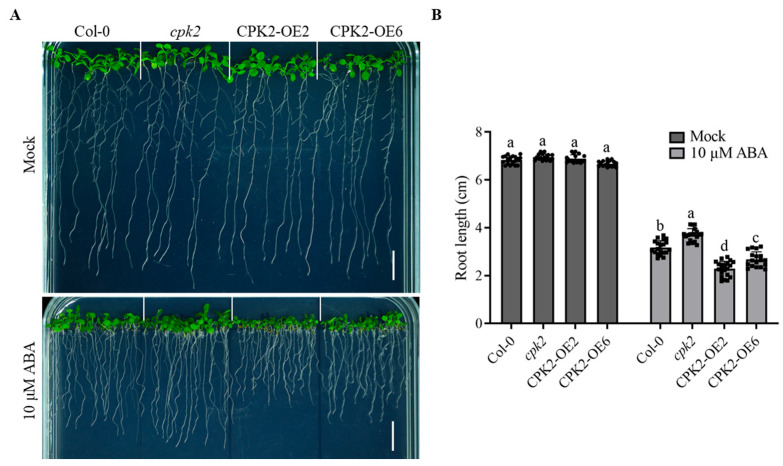
CPK2 enhances the sensitivity of root growth to ABA. (**A**) Root phenotypes of WT, *cpk2* mutants, and two overexpression lines on normal medium or medium supplemented with 10 μM ABA. (**B**) Primary root length was measured at 10 d after germination. Data are presented as means ± SD (*n* = 20). Statistics were performed using two-way ANOVA followed by Tukey’s multiple comparisons test within the same treatment. Different lowercase letters (a, b, c, d) indicate significant differences among genotypes (*p* < 0.05). Dots and squares represent individual root length measurements, with symbol type used to distinguish experimental conditions. Scale bars, 1 cm.

To investigate the role of CPK2 in ABA signaling during early seedling development, we obtained a T-DNA insertion mutant, *cpk2* (SALK_036166), and generated *CPK2*-overexpressing lines under the control of the CaMV 35S promoter. Two independent *CPK2*-overexpressing lines, *CPK2-OE2* and *CPK2-OE6*, were selected based on RT-qPCR analysis (Supplementary Figure S1). Seeds of wild type (Col-0), *cpk2*, and the two *CPK2*-overexpressing lines, *CPK2-OE2* and *CPK2-OE6*, were sown on 1/2 MS medium supplemented with 10 μM ABA. No significant differences in root growth were observed among genotypes on ABA-free medium. However, in the presence of ABA, the *cpk2* mutant exhibited reduced sensitivity to ABA, whereas the *CPK2*-overexpressing lines showed enhanced sensitivity compared to the wild type (Figure 1A,B).

### 3.2. CPK2 Physically Interacts with ABI5

**Figure 2:**
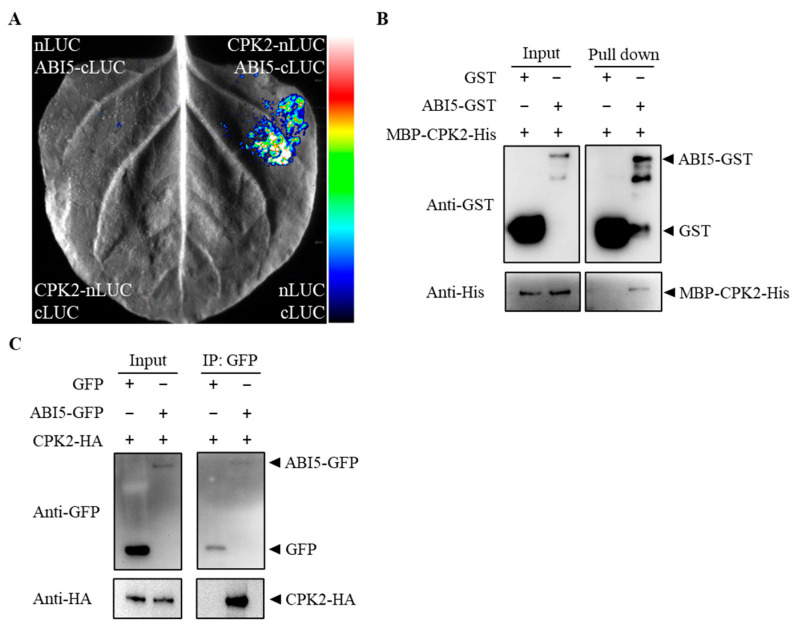
CPK2 physically interacts with ABI5. (**A**) Split LUC assay. CPK2-nLUC and ABI5-cLUC constructs were transiently expressed in *N. benthamiana* leaves. The pseudocolor scale represents LUC fluorescence intensity, reflecting the strength of protein interaction, with blue indicating low, green to yellow medium, and red high signal intensity. (**B**) GST pull-down analysis. MBP-CPK2-His was mixed with GST-ABI5 or GST and immobilized on GST beads. After washing, the eluted proteins were subjected to immunoblot analysis with anti-GST and anti-His antibodies, respectively. (**C**) Co-IP assays showing that CPK2 associates with ABI5 in vivo. CPK2-HA was co-expressed with ABI5-GFP or GFP in *N. benthamiana* leaves, and then the total proteins were extracted and incubated with GFP beads (MBL). The total and precipitated proteins were subjected to immunoblotting with GFP and HA antibodies, respectively.

As a central transcription factor in the ABA signaling pathway, ABI5 plays a crucial role in regulating ABA-responsive gene expression during seed dormancy, germination, and seedling growth [15]. To investigate whether CPK2 is involved in ABA signaling through interaction with ABI5, we first performed a luciferase complementation imaging (LCI) assay. CPK2 was fused to the N-terminal half of luciferase (nLUC), and ABI5 was fused to the C-terminal half (cLUC). The constructs were co-expressed in *N. benthamiana* leaves. A strong luminescent signal (LUC activity) was observed upon co-expression of CPK2 and ABI5, whereas no signal was detected in the negative controls, indicating that CPK2 interacts with ABI5 in planta (Figure 2A). To determine whether this interaction is direct, we conducted an in vitro GST pull-down assay. Recombinant GST-ABI5, but not GST alone, efficiently pulled down His-CPK2, confirming a direct physical interaction between the two proteins (Figure 2B). To further validate this interaction in vivo, we conducted co-immunoprecipitation (Co-IP) experiments. CPK2-HA and ABI5-GFP were co-expressed in *N. benthamiana* leaves, and GFP-tagged proteins were immunoprecipitated using GFP-Trap beads. Immunoblotting with anti-HA and anti-GFP antibodies showed that CPK2-HA was specifically co-precipitated with ABI5-GFP, but not with free GFP (Figure 2C).

### 3.3. CPK2 Phosphorylates ABI5

**Figure 3:**
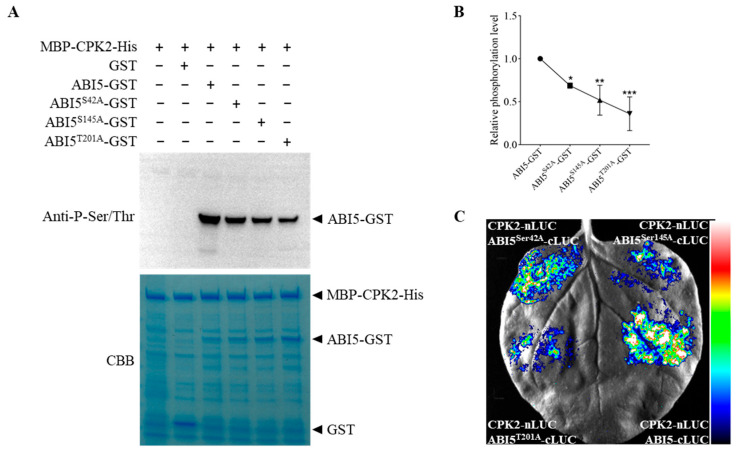
CPK2 phosphorylates ABI5. (**A**) In vitro phosphorylation assay showing the phosphorylation of ABI5 at Ser 42, Ser 145, and Thr 201 by CPK2. Phospho-dead variants of ABI5 were generated by mutation of serine/threonine residues at 42, 145, and 201 to alanine (**A**). (**B**) The relative phosphorylation signals of ABI5, as shown in (**A**), were analyzed using Image J (version 1.51j8). Statistics were performed using one-way ANOVA followed by Tukey’s multiple comparisons test between samples. * *p* < 0.05, ** *p* < 0.01, *** *p* < 0.001. Data are mean ± SD. (**C**) Split LUC assay. CPK2-nLUC and different variant ABI5-cLUC constructs were transiently expressed in *N. benthamiana* leaves. The pseudocolor scale represents LUC fluorescence intensity, reflecting the strength of protein interaction, with blue indicating low, green to yellow medium, and red high signal intensity.

Calcium-dependent protein kinases (CPKs) are known to phosphorylate downstream substrates at serine or threonine residues, thereby regulating various aspects of plant growth and stress responses [28]. To determine whether CPK2 can phosphorylate ABI5, we performed in vitro phosphorylation assays using purified recombinant proteins. These assays confirmed that CPK2 directly phosphorylates ABI5 (Figure 3A). Previous studies have shown that the Ser42, Ser145, and Thr201 residues of ABI5 are phosphorylated both in vivo and in vitro [19,22,44]. To investigate whether these residues serve as phosphorylation targets of CPK2, we generated site-directed mutants in which Ser42, Ser145, and Thr201 were individually mutated to alanine (Ala), mimicking a non-phosphorylation state. When tested in in vitro phosphorylation assays, each mutant showed significantly reduced phosphorylation by CPK2 compared to wild-type ABI5 (Figure 3A,B), indicating that these residues contribute to CPK2-mediated phosphorylation. Notably, luciferase complementation imaging (LCI) assays showed reduced interaction between CPK2 and ABI5 in these mutants. Specifically, when ABI5^Ser42^-cLUC, ABI5^Ser145^-cLUC, or ABI5^Thr201^-cLUC was co-expressed with CPK2-nLUC in *N. benthamiana* leaves, LUC activity was markedly decreased compared to wild-type ABI5 (Figure 3C).

### 3.4. CPK2 Enhances ABA-Induced ABI5 Expression and Stability

**Figure 4:**
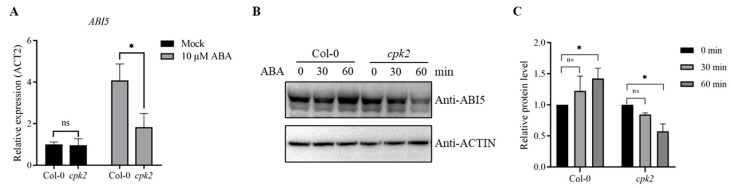
CPK2 is involved in the regulation of ABA-induced *ABI5* gene expression and ABI5 protein stability. (**A**) Expression of *ABI5* in Col-0 and *cpk2* mutants. Seven-day-old seedlings of different genotypes were treated with or without 10 μM ABA for 3 h, and *ABI5* gene expression was detected by qRT-PCR. Values are means ± SD, *n* = 3. Statistics were performed using Student’s *t*-test (* *p* < 0.05). (**B**) ABI5 protein abundance in Col-0 and *cpk2* mutants after ABA treatment for different times. Seven-day-old Col-0 and *cpk2* mutant seedlings were first treated with 10 μM ABA and then harvested at the indicated times after treatment. The ABI5 protein was detected by western blot assay using ABI5 antibodies. Anti-Actin was used as a sample loading control. (**C**) Quantification of protein intensities from (**B**), normalized to wild-type control (set as 1.0). Statistics were performed using one-way ANOVA followed by Tukey’s multiple comparisons test between samples. * *p* < 0.05; ns indicates no significant difference. Data are presented as means ± SD (*n* = 3).

To explore the molecular mechanism by which CPK2 regulates ABA signaling during seedling growth, we investigated whether CPK2 modulates the expression and accumulation of ABI5. Seven-day-old seedlings of Col-0 and *cpk2* mutants were transferred to 1/2 MS medium supplemented with or without 10 μM ABA for 3 h, followed by qRT-PCR analysis. Under control conditions, *ABI5* transcript levels were comparable between Col-0 and *cpk2*. ABA treatment strongly induced *ABI5* expression in both genotypes, but this induction was significantly attenuated in the *cpk2* mutant compared to Col-0 (Figure 4A). We further analyzed the influences of ABA on ABI5 accumulation under Col-0 and *cpk2* mutant backgrounds. Col-0 and *cpk2* mutant seedlings were treated with 10 μM ABA for 0, 30, and 60 min, and the samples were harvested at the indicated time for the western blot assay. The results indicated that ABI5 protein levels increased after ABA treatment in Col-0 seedlings, whereas ABA treatment significantly reduced the ABI5 accumulation in the *cpk2* mutant (Figure 4B).

### 3.5. Genetic Relationship Between CPK2 and ABI5

**Figure 5:**
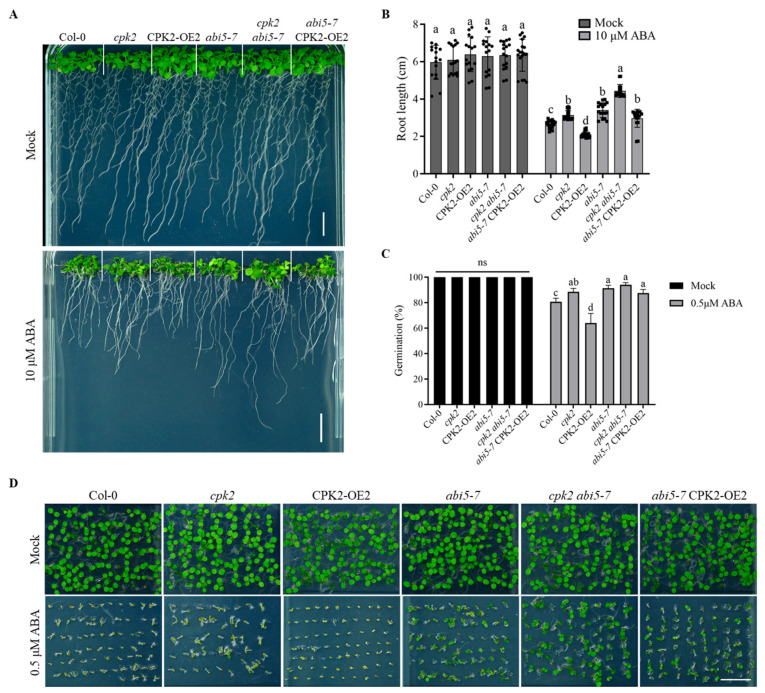
CPK2 and ABI5 genetically interact to regulate ABA responses in *Arabidopsis*. (**A**) Phenotypic analyses of the root growth of different genotypes. Seeds were germinated and grown vertically on 1/2 MS medium or 1/2 MS medium with 10 μM ABA for 10 days. (**B**) Primary root length was measured from (**A**). Data are presented as means ± SD (*n* = 3). Statistical analysis was performed using two-way ANOVA followed by Tukey’s multiple comparisons test within the same treatment. Different lowercase letters indicate significant differences among genotypes (*p* < 0.05). Dots and squares represent individual root length measurements, with symbol type used to distinguish experimental conditions. Scale bars, 1 cm. (**C**) Germination rate was recorded from (**D**). Data are presented as means ± SD (*n* = 3). Statistical analysis was performed using two-way ANOVA followed by Tukey’s multiple comparisons test within the same treatment. Different lowercase letters indicate significant differences among genotypes (*p* < 0.05); ns indicates no significant difference. Dots and squares represent individual root length measurements, with symbol type used to distinguish experimental conditions. Data are presented as means ± SD (*n* = 3). Scale bars, 1 cm. (**D**) Seed germination phenotypes. Seeds were sown on 1/2 MS medium with or without 0.5 μM ABA and grown for 3 days.

Given that ABI5 is a known positive regulator of ABA signaling, we sought to determine whether CPK2 acts synergistically with ABI5 in modulating ABA responses. To investigate the genetic relationship between CPK2 and ABI5, we generated double mutants by crossing *abi5-7* with either the *cpk2* mutant or the CPK2-overexpressing line, *CPK2-OE2*, resulting in *cpk2abi5-7* and *abi5-7CPK2-OE2*, respectively. Seeds of these genotypes, as well as the wildtype Col-0, were sown on the 1/2 MS medium supplemented with or without 10 μM ABA, and root lengths were measured after 10 days of treatment. No significant differences in root length were observed among the genotypes on the ABA-free medium. ABA treatment inhibited root growth in all genotypes, but to varying degrees. The *CPK2-OE2* line exhibited the greatest sensitivity (strongest inhibition), whereas the *cpk2abi5-7* double mutant showed the least sensitivity (weakest inhibition). Notably, the *cpk2*, *abi5-7*, and *abi5-7CPK2-OE2* lines all displayed reduced ABA sensitivity compared to Col-0 (Figure 5A,B). To assess whether this ABA response phenotype extends to seed germination, seeds of Col-0, *cpk2*, *abi5-7*, *CPK2-OE2*, *cpk2abi5-7*, and *abi5-7CPK2-OE2* were sown on 1/2 MS medium supplemented with or without 0.5 μM ABA. Germination rates were recorded after 3 days of stratification. Under control conditions, all genotypes exhibited complete germination with no significant differences. In the presence of ABA, the *cpk2* mutant displayed reduced ABA sensitivity compared to Col-0 but was more sensitive than *abi5-7* (higher germination rate than Col-0 and lower than *abi5-7*). In contrast, *CPK2-OE2* seeds showed enhanced ABA sensitivity (lower germination rate than Col-0). The *cpk2abi5-7* double mutant exhibited less sensitivity to ABA than either *cpk2* or *abi5-7* single mutants, and the *abi5-7CPK2-OE2* line was less sensitive than both Col-0 and *CPK2-OE2* (higher germination rate than either genotype) (Figure 5C,D).

## 4. Discussion

Abscisic acid (ABA) plays critical roles in regulating plant growth and development under both normal conditions and adverse environmental stresses, including drought, high salinity, and cold. Upon exposure to these adverse conditions, ABA level rises rapidly, functioning as a central hormonal signal that initiates a broad spectrum of adaptive physiological responses [12,45,46,47]. Among the most critical developmental processes regulated by ABA are seed germination and root growth, which are vital for nutrient and water uptake and for environmental adaptation [48].

ABI5 (ABA INSENSITIVE 5), a key transcription factor in the ABA signaling cascade, orchestrates plant responses throughout the life cycle and in response to environmental cues. As a central regulatory hub, ABI5 integrates ABA signals with developmental programs and other phytohormone pathways, fine-tuning stress adaptation by regulating the expression of numerous downstream genes. The activity of ABI5 is tightly controlled through multiple regulatory layers, including transcriptional induction, protein stability, subcellular localization, and post-translational modifications (PTMs), ensuring precise responsiveness to dynamic ABA signals [49,50,51,52,53]. Among PTMs, phosphorylation is a major mechanism modulating ABI5 function. Several kinases have been shown to phosphorylate ABI5 at distinct residues, leading to divergent functional outcomes [17]. For instance, SnRK2s, core components of the ABA signaling pathway, phosphorylate ABI5 at Ser42, Ser145, and Thr201, promoting its stability and transcriptional activity under stress [19,22,44]. Phosphorylation at these sites can create docking motifs for downstream effectors or prevent recognition by E3 ubiquitin ligases such as KEG and COP1, thereby delaying proteasomal degradation [50,54]. Conversely, MPK3-mediated phosphorylation promotes ABI5 inactivation [55]. This complex phosphorylation network allows fine-tuned control of ABI5 abundance and activity in response to internal and external stimuli. Despite this progress, the role of calcium-dependent signaling in modulating ABI5 remains incompletely defined, particularly in the context of cross-talk between Ca^2+^ and ABA pathways.

Calcium-dependent protein kinases (CPKs/CDPKs) are key mediators that translate calcium transients into phosphorylation events during stress responses. In *Arabidopsis thaliana*, the CPK family comprises 34 members that exhibit diverse roles in growth, development, and stress adaptation, among which CPK2 has been identified as an ER-localized kinase [30,56]. Multiple CPKs—including CPK3, CPK4, CPK6, CPK9, CPK10, CPK11, and CPK32, are involved in ABA signaling [32,33,35,36,38,57]. Functional redundancy among CPKs suggests a robust and layered regulatory network that ensures signaling fidelity under fluctuating conditions. Beyond Arabidopsis, emerging evidence supports the evolutionary conservation of CPK functions in ABA signaling, such as BnaCPK5 in Brassica napus and MdCPK4 in Malus domestica, which also function in ABA signaling through phosphorylation of key regulators (ABFs and PYLs) [58,59]. Such functional redundancy implies that CPK2 may share overlapping roles with other CPKs in certain aspects of ABA signaling, thereby enhancing the robustness and precision of the plant’s response to stress.

In this study, we provide compelling evidence that *cpk2* mutants exhibit ABA-insensitive phenotypes, whereas CPK2-overexpressing lines display heightened ABA sensitivity during seed germination and root growth (Figure 1 and Figure 5), establishing CPK2 as a positive regulator of ABA signaling. Through comprehensive genetic, biochemical, and molecular analyses, we reveal that CPK2 increases the sensitivity of seed germination and root growth to ABA by promoting ABA-mediated *ABI5* expression (Figure 1 and Figure 4A). Furthermore, CPK2 also enhanced the ABA-mediated ABI5 stability by directly interacting with ABI5 and phosphorylating it at S42, S145, and T201 (Figure 2, Figure 3 and Figure 4B). Our findings suggest that CPK2 may act in parallel or cooperatively with SnRK2s to reinforce ABI5 activation under calcium-elevating stress conditions, such as drought or high salinity, where Ca^2+^ serves as a secondary messenger. From an applied perspective, the CPK2–ABI5 module offers a promising target for improving abiotic stress tolerance in crops. Unlike constitutive overexpression of ABI5, which often leads to severe growth penalties, modulating CPK2 activity or engineering phosphorylation-mimic variants of ABI5 could enable inducible stress responses with minimal fitness cost.

Nonetheless, several limitations warrant further investigation. First, while our in vitro kinase assays demonstrate that CPK2 can phosphorylate ABI5 at S42, S145, and T201, the physiological relevance of these phosphorylation events in vivo requires further validation. The kinase activity and substrate specificity observed in vitro may not fully recapitulate the dynamic and context-dependent regulation occurring in living cells. Second, many of our protein–protein interaction studies were performed in the *N. benthamiana* transient expression system. Although widely used and informative, this heterologous system may not fully recapitulate the native interaction landscape in Arabidopsis, due to differences in post-translational modifications, subcellular environments, or the absence of endogenous regulatory factors.

**Figure 6:**
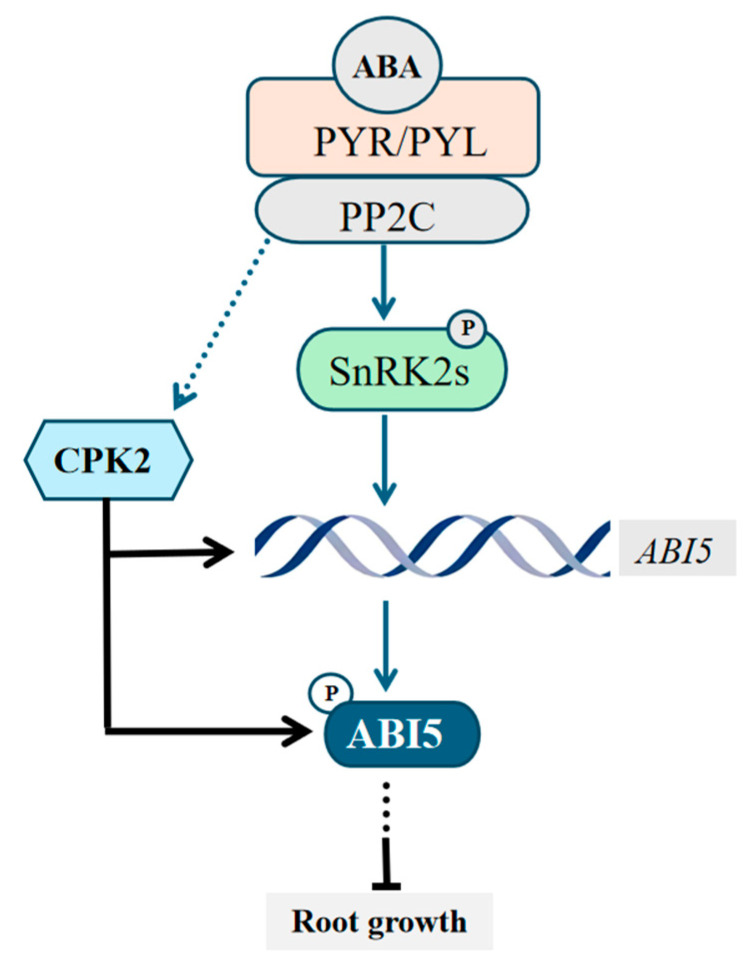
A proposed model of CPK2 functions in ABA-mediated seed germination and root growth inhibition in *Arabidopsis*. ABA binds to PYL/PYR receptors, which inhibit PP2Cs and lead to the activation of SnRK2s. Activated SnRK2s phosphorylate downstream effectors such as ABI5 to mediate ABA signaling. CPK2, a calcium-dependent protein kinase (CPK) family member, activated by unknown upstream signals, enhances ABA-mediated *ABI5* expression and the ABI5 protein stability by interacting and phosphorylating it, as a result, exacerbates the ABA inhibition of seed germination and root growth.

In summary, our results provide evidence that CPK2, as a positive regulator in the ABA signaling pathway, is involved in the regulation of seed germination and root growth by enhancing both the expression and stability of ABI5 in *Arabidopsis* (Figure 6). Together, these findings expand our understanding of the molecular mechanisms linking calcium signaling to ABA responses and highlight the importance of kinase-mediated regulation of key transcription factors in stress adaptation.

## 5. Conclusions

In conclusion, this study elucidates a critical role for CPK2 in modulating ABA signaling during seed germination and root growth by directly targeting the key transcription factor ABI5. This CPK2-ABI5 module represents a critical regulatory node in ABA signaling. These findings provide a potential target for improving abiotic stress tolerance in crops through biotechnological approaches. Further validation in crop species will be essential to translate these findings into agricultural applications.

## Data Availability

All data and materials that support the findings of this study are included in the manuscript or in the Supplementary Materials.

## Supplementary Materials

The following supporting information can be downloaded at https://www.mdpi.com/article/10.3390/plants14172671/s1: Figure S1: Molecular characterization of the *cpk2* mutant and transgenic lines. (A) Schematic diagram showing the T-DNA insertion site in the *cpk2* mutant. (B) Quantitative RT-PCR analysis of *CPK2* gene expression in wild-type (Col-0), *cpk2* mutant, CPK2-overexpressing line (*CPK2-OE2*, *CPK2-OE6*) and *abi5-7CPK2-OE2*. (C) Quantitative RT-PCR analysis of ABI5 gene expression in wild-type (Col-0), *abi5-7* mutant, *cpk2abi5-7,* and *abi5-7CPK2-OE2*; Figure S2: Schematic diagrams of the constructs used in this study. (A) pGWB505-CPK2-EGFP: A plant overexpression vector in which the CPK2 coding sequence is fused in-frame to the N-terminus of EGFP under the control of the CaMV 35S promoter. (B) pMAL-MBP-CPK2-His: A bacterial expression vector for production of recombinant CPK2 protein. (C,D) pGWB414-CPK2-HA and pGWB405-ABI5-EGFP: Used for Co-IP assays. (E) pCambia1300-CPK2-nLUC: Used in LCI assays; Table S1: Primers used in this study.
