# Supplementary material for: CPK2 Enhances ABA Sensitivity in Seed Germination and Root Growth by Promoting ABA-Induced ABI5 Expression and ABI5 Protein Stability
Source: Plants (Basel). 2025 Aug 27;14(17):2671. doi: 10.3390/plants14172671 (PMC12430662; doi:10.3390/plants14172671)
Supplement: Supplementary file 1 [file plants-14-02671-s001.zip › Supplemental Table S1.pdf]

Supplemental Table S1. Primer Sequences Used in this study.

Primer name	Primer sequence (5'-3')	Purpose
cpk2 SALK_036166-LP	TTAAGCGGAGTTCCTCCTTTC	genotyping
cpk2 SALK_036166-RP	CTTCAACACCAAACTCTTCGC	genotyping
LBb1.3	ATTTTGCCGATTTCGGAAC	genotyping
CPK2-qF	TCAAAGACCAATCCCGAAACTA	qPCR
CPK2-qR	GGCTTGGATTCAGACTTTGTTT	qPCR
ACTIN2-qF	TTGACTACGAGCAGGAGATGG	qPCR
ACTIN2-qR	ACAAACGAGGGCTGGAACAAG	qPCR
ABI5-GFP-F	gagaacacgggggacATGGTAACTAGAGAAAC GAAG	Co-IP
ABI5-GFP-R	gctcaccattctagaGAGTGGACAACTCGGGTTC	Co-IP
CPK2-HA-F	AGAACACGGGGGACTCTAGAATGGGTA ATGCTTGCGTTGGAC	Co-IP
CPK2-HA-R	ATATGGATAGGATCCATGTTTCAGAGAAA TGCTAATG	Co-IP
CPK2-nLUC-F	CGGTACCCGGGATCCATGGGTAATGCTT GCG	LCI
CPK2-nLUC-R	CGAGATCTGGTCGACATGTTTCAGAGAA ATGCTAATG	LCI
MBP-CPK2-His-F	ACTAATTCGAGCTCCATGGGTAATGCTTG CGTTG	Pull-down
MBP-CPK2-His-R	GTGGTGGTGCTCGAGATGTTTCAGAGAA ATGCTAATGCTG	Pull-down
ABI5^{S42A}-F	GACAATCCgCTATCTACTCAT	Mutagenesis
ABI5 ^{S42A} -R	ATGAGTAGATAGcGGATTGTC	Mutagenesis
ABI5^{S145A}-F	CGACAAGGCgCTTTGACACTT	Mutagenesis
ABI5^{S145A}-R	AAGTGTCAAAGcGCCTTGTCG	Mutagenesis
ABI5^{T201A}-F	AGACAACCGgCTTTTGGAGAG	Mutagenesis
ABI5^{T201A}-R	CTCTCCAAAAGcCGGTTGTCT	Mutagenesis
