# Supplementary figures and images for: CPK2 Enhances ABA Sensitivity in Seed Germination and Root Growth by Promoting ABA-Induced ABI5 Expression and ABI5 Protein Stability
Source: Plants (Basel). 2025 Aug 27;14(17):2671. doi: 10.3390/plants14172671 (PMC12430662; doi:10.3390/plants14172671)

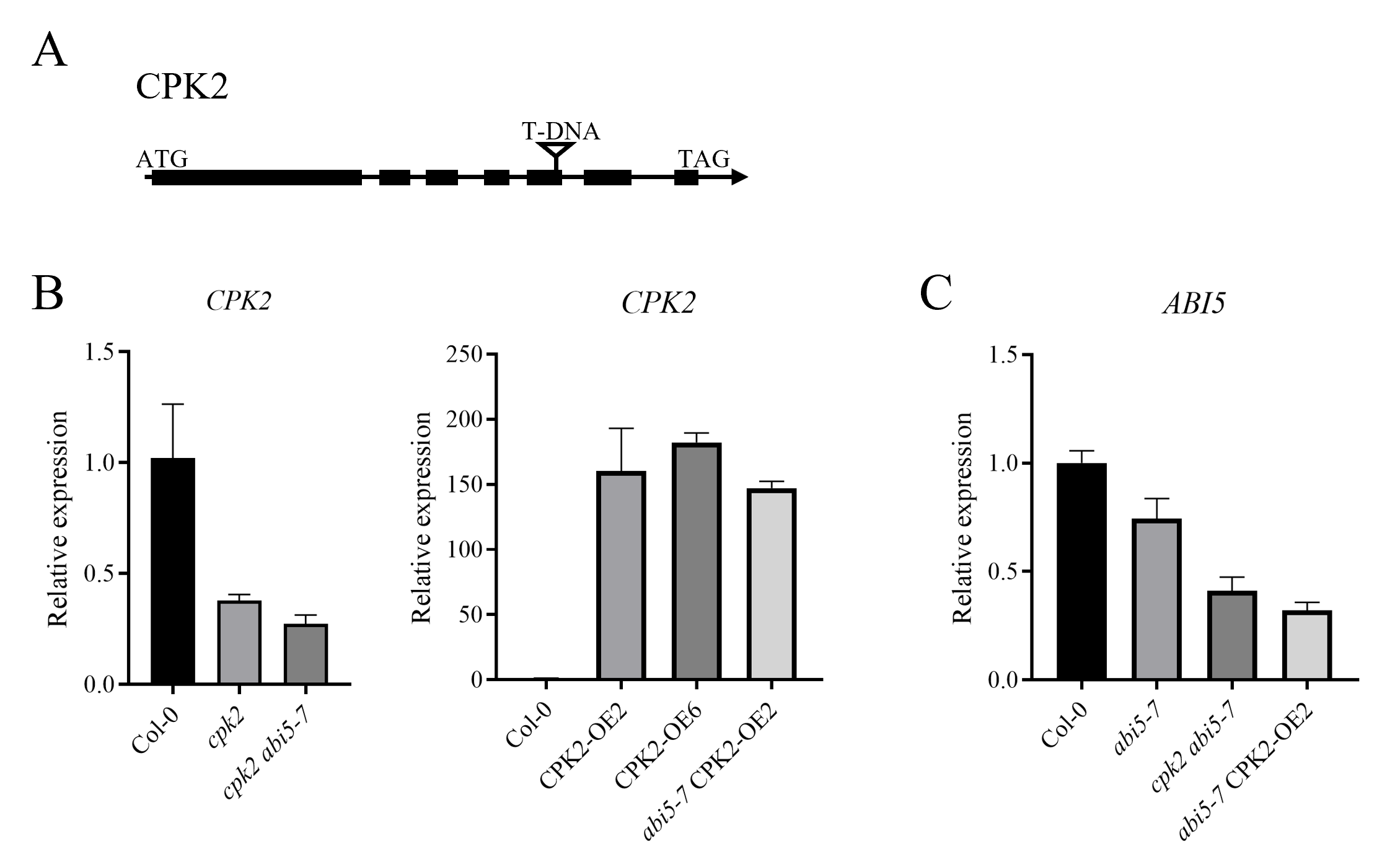

Supplement: Supplementary file 1 [file plants-14-02671-s001.zip › Figure S1.tif]

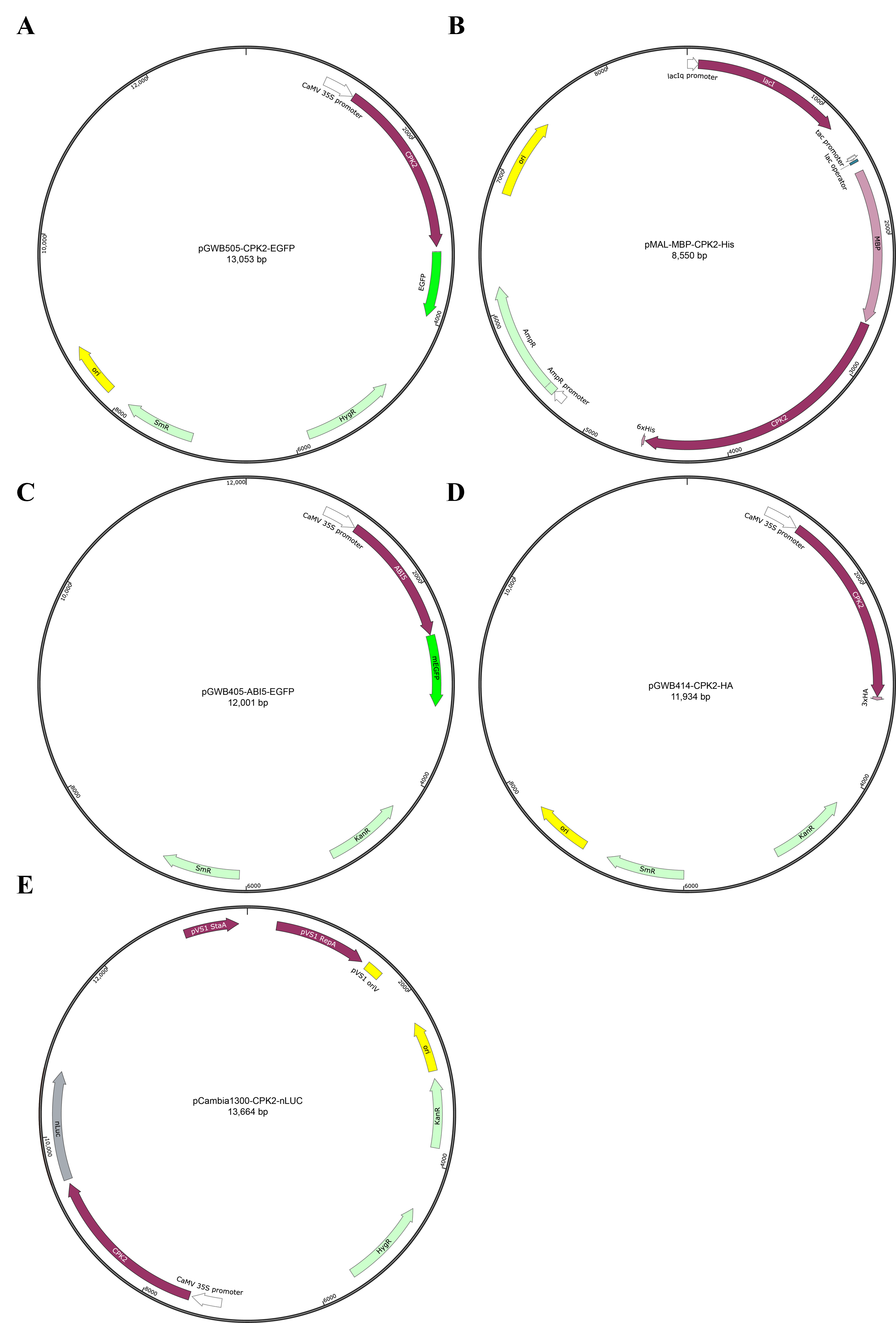

Supplement: Supplementary file 1 [file plants-14-02671-s001.zip › Figure S2.tif]
